# Unambiguous Imaging of Static Scenes and Moving Targets with the First Chinese Dual-Channel Spaceborne SAR Sensor

**DOI:** 10.3390/s17081709

**Published:** 2017-07-25

**Authors:** Tingting Jin, Xiaolan Qiu, Donghui Hu, Chibiao Ding

**Affiliations:** 1 Key Laboratory of Technology in Geo-spatial Information Processing and Application Systems, Institute of Electronics, Chinese Academy of Sciences, Beijing 100190, China; jintingting13@mails.ucas.ac.cn (T.J.); dhhu@mail.ie.ac.cn (D.H.); cbding@mail.ie.ac.cn (C.D.); 2 School of Electronic, Electrical and Communication Engineering, University of the Chinese Academy of Sciences, Beijing 100049, China

**Keywords:** synthetic aperture radar (SAR), high-resolution and wide-swath (HRWS), channel error estimation, reconstruction algorithm, unambiguous imaging

## Abstract

Multichannel synthetic aperture radar (SAR) is a breakthrough given the inherent limitation between high-resolution and wide-swath (HRWS) faced with conventional SAR. This paper aims to obtain unambiguous imaging of static scenes and moving targets with the first Chinese dual-channel spaceborne SAR sensor. We propose an integrated imaging scheme with the dual-channel echoes. In the imaging scheme, the subspace-based error estimation algorithm is first applied to the spaceborne multichannel SAR system, followed by the reconstruction algorithm prior to imaging. The motion-adapted reconstruction algorithm for moving target imaging is initially achieved with the spaceborne multichannel SAR system. The results exhibit an effective suppression of azimuth ambiguities and false targets with the proposed process. This paper verifies the accuracy of the subspace-based channel error estimator and the feasibility of the motion-adapted reconstruction algorithm. The proposed imaging process has prospects for future HRWS SAR systems with more channels.

## 1. Introduction

Spaceborne synthetic aperture radar (SAR) sensors are an increasingly influential tool for remote sensing of the Earth. Demands for better observation performance increased the requirements for both spatial resolution and swath width, which are contradictory for the system design of conventional SAR. Higher azimuth resolution requires higher pulse repetition frequency (PRF), while wider swath requires lower PRF, creating an irreconcilable conflict. Fortunately, the multichannel SAR system has been proposed to obtain high-resolution and wide-swath (HRWS) imaging simultaneously by splitting the antenna into multiple receive channels in azimuth [1,2,3,4]. Low PRF is transmitted to obtain an unambiguous wide swath, then echoes of multiple receivers are combined to improve the azimuth resolution and eliminate azimuth ambiguities.

The feasibility of this mode was first verified by the German satellite TerraSAR-X launched in 2007 [3], then by the Japanese satellite AlOS-2 launched in 2014 [4], which both contain dual receive channels. The launch of the Chinese satellite Gaofen-3 in 2016 marks the first Chinese dual-channel spaceborne SAR sensor. Different from traditional single-channel SAR, signal processing of multichannel echoes faces several difficulties. Firstly, channel imbalances are inevitable due to the multichannel system. Secondly, the echoes of multichannel SAR are always non-uniformly sampled in azimuth. In addition, false targets will arise in the imaging results of the moving targets if addressed along with the static scenes. The velocity of the moving target can be divided into radial and along-track components, which are respectively perpendicular and parallel to the flight direction of the satellite platform. The along-track velocity of the moving target is far smaller than the satellite velocity, thus can be ignored. The radial velocity is the primary cause of false targets, and should thus be estimated beforehand in order to obtain unambiguous imaging.

Various algorithms used to estimate channel mismatches have been proposed in recent years. The azimuth cross correlation method proposed in [5], the orthogonal subspace method (OSM) and the signal subspace comparison method (SSCM) proposed in [6] are representative algorithms. These methods can achieve accurate estimation of amplitude and phase errors among different receive channels and their efficiencies have been validated in [7]. However, detailed analysis of the mechanism of channel imbalances for on-orbit SAR systems is lacking. Moreover, a necessary step remains to be investigated to complete the spaceborne experiment of error estimation with the OSM. As for a solution to the non-uniform sampling problem, recent years have witnessed plenty of research on the digital beaming forming (DBF) and unambiguous imaging of stationary scenes. A reconstruction algorithm was proposed to recover the unambiguous signal in [8]. Then Kim et al. gave the first spaceborne demonstration of channel reconstruction with TerraSAR-X dual receive channel (DRC) mode [9]. In general, previous studies achieved good applications of unambiguous reconstruction on multichannel SAR imaging. Nevertheless, the space-time spectrum of the moving target has a linear offset from the static ones; false targets exist if moving target signals are processed with the reconstruction algorithm in [8,9]. Hence, the Doppler spectrum reconstruction is more complicated for application to a moving target. Novel multichannel signal reconstruction algorithms for moving targets were proposed in [10,11], where a motion-adapted HRWS reconstruction was proposed in [10] and investigated in [11]. 

In order to obtain the motion-adapted reconstruction filter, the radial velocity of the moving target should be accurately estimated [12,13,14,15,16,17,18,19,20]. In [12,13], the along-track interferometry (ATI) method was applied to the RadarSAT-2 and TerraSAR-X satellites to estimate the radial velocity and obtained good results. References [14,15,16] proposed some additional velocity estimation algorithms conducted in image domain, namely the Displaced Phase Center Array (DPCA) technique, the relative residue of DPCA (RR-DPCA) method, and the application of eigen-decomposition of the sample multi-channel covariance matrix. The radial velocity can also be estimated in the signal domain. In [17], the radial velocity is estimated by measuring the azimuth offset after multichannel reconstruction and imaging. However, this method needs the additional process of imaging and distinguishing between false targets and the real one, and the measurement error will influence the estimation accuracy. In [18], the radial velocity estimation is transformed to the Doppler centroid estimation based on the multilook cross-correlation, but it needs the ambiguity number resolving approach. In [19,20], the radial velocity estimation issue is transformed to the cone angle estimation according to their relationship. These methods are accurate, with the shortcoming of needing additional searching process.

In this paper, we propose an integrated unambiguous imaging algorithm, moving target estimation, and imaging algorithm with Chinese Gaofen-3 dual receive channel (DRC) mode. We realize the first successful application of the OSM in the spaceborne experiment of channel error estimation. Moreover, we achieve the moving target’s velocity estimation and unambiguous imaging of the first Chinese dual-channel spaceborne SAR sensor. The unambiguous imaging algorithm is divided into three steps: step one, the subspace-based estimator is applied to estimate the channel mismatch and the estimated errors are compensated; step two, the reconstruction algorithm is applied to obtain equivalent single-channel echo; and step three, the chirp scaling (CS) algorithm is applied to obtain the unambiguous images. As the reconstruction filter is mismatched for the moving targets, the signals of moving targets are still non-uniformly sampled, resulting in false targets along the azimuth around the real one. Focused imaging of the moving target requires accurate estimation of the radial velocity. We extracted the echoes of moving targets after range compression and applied an imaging algorithm to each channel. Then the ATI method is applied to estimate the radial velocity. After that, the moving target signals are reconstructed with a motion-adapted reconstruction filter. Finally, the moving target is imaged separately to suppress false targets. The primary goal of this paper is to demonstrate the effectiveness of the OSM and the reconstruction algorithm on the dual-channel spaceborne SAR sensor, and the feasibility of unambiguous imaging of the moving target, providing reference for future HRWS SAR systems with more channels.

This paper begins in Section 2 with an overview of Gaofen-3 DRC mode, with a discussion of sources of channel mismatches and expression of signal models. In Section 3, the imaging process including error estimation, unambiguous reconstruction, and the radial velocity estimation is explained. Experimental results are presented in Section 4, followed by some discussion in Section 5. Section 6 draws conclusions and discusses future perspectives.

## 2. Gaofen-3 DRC Mode and Signal Model

### 2.1. Gaofen-3 DRC Mode

Gaofen-3 DRC mode, or ultra-fine stripmap mode, is one of the new features of the Chinese spaceborne SAR sensor. This system improves the swath width in stripmap mode without degradation of azimuth resolution.

**Figure 1:**
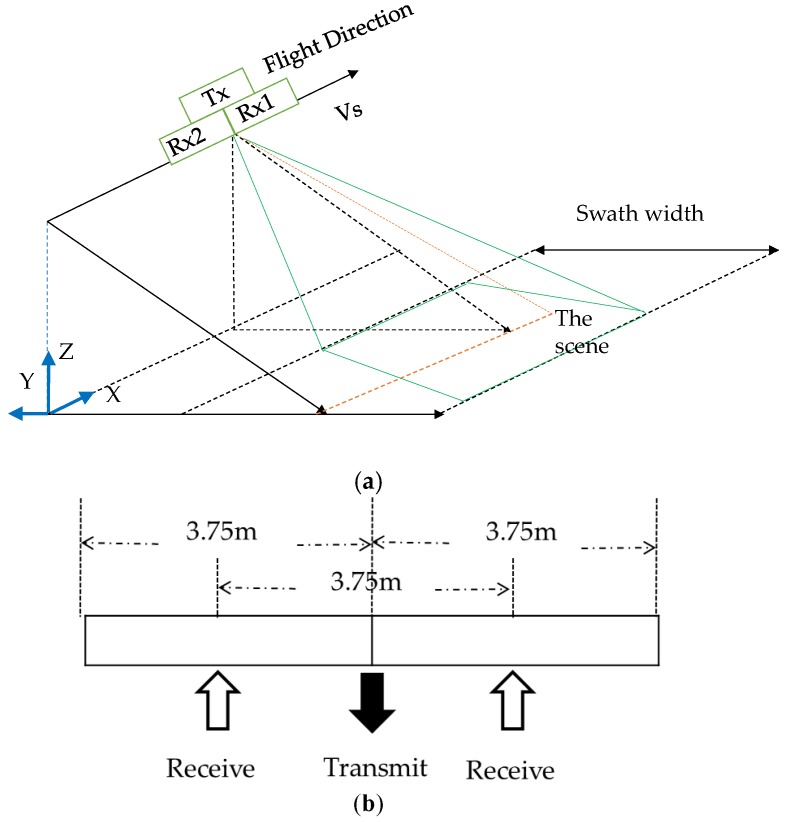

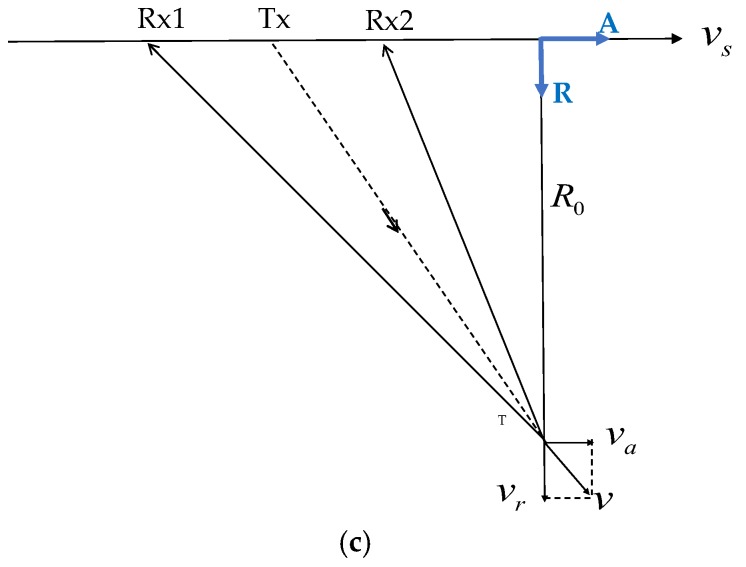
Illustration of Gaofen-3 DRC mode: (**a**) geometric model of the system; (**b**) transmitting and receiving of the antenna; (**c**) geometry in the slant-range plane.

Figure 1 is a brief illustration of the geometric model of the Chinese Gaofen-3 DRC mode. The antenna transmits chirp signals at the center (Tx), and two separate channels (Rx1 and Rx2) in azimuth receive echoes simultaneously, as shown in Figure 1a. Figure 1b is the illustration of transmitting and receiving antennae, where the aperture size and distance between two receive channels are 3.75 m. If there is a moving target in the detected scene, the geometry of the platform and the moving target in the slant-range plane occurs, as shown in Figure 1c. According to the geometrical relationship, the velocity of the moving target can be divided into the along-track and radial velocities. Thanks to dual receive channels, Gaofen-3 ultra-fine stripmap mode can achieve approximately 3 m resolution in azimuth, with a PRF of less than 2000 Hz. It is difficult to obtain such spatial resolution with the same PRF for the conventional stripmap SAR. 

**Figure 2:**
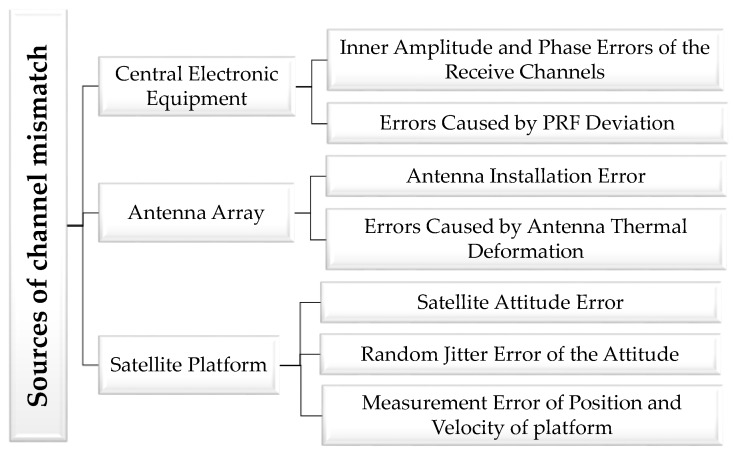
Sources of channel mismatch.

There are three main sources of errors: satellite platform, antenna array, and central electronic equipment. Detailed classification of error sources is illustrated in Figure 2. According to the characteristics of the errors, they can be divided into three categories: constant errors, random errors, and high-frequency jittered errors. Constant errors can be compensated for through inner calibration, and high-frequency jittered errors can be neglected because of their harmonic characteristics. Of all the sources of errors, measure errors of platform velocity and PRF deviation cause non-uniform sampling, making reconstruction indispensable. Other random errors result in magnitude and phase imbalances, which should be estimated and compensated for before imaging.

### 2.2. Signal Model

The symbols of the parameters used in this paper are listed as follows:
mindex of receive channels, m = 1,2;τ, ηrange time and azimuth time, respectively;$\Gamma_{m}$, $\zeta_{m}$total random amplitude error and phase error of the
*m*-th channel, respectively;h(τ), g(η)range antenna pattern and azimuth antenna pattern, respectively;$v_{s}$platform velocity;*d*distance between adjacent receive channels;$x_{m}$azimuth center of
*m*-th receive channel;$\Delta x_{m}$measurement error of azimuth center of the antenna;$v_{a},v_{r}$along-track and radial velocity of the moving target, respectively;λwavelength of the radar signal;σ(x,y)point scattering coefficient at
(x,y,0); $\sigma_{t}$complex scattering coefficient of the target;$T_{s}$synthetic aperture time;$T_{p}$, $K_{r}$pulse width and chirp rate of transmitted signal, respectively; and$f_{p}$pulse repetition frequency (PRF).

#### 2.2.1. Signal Model of Static Scene

The geometric model of DRC mode is shown in Figure 1a. The SAR system transmits chirp signals at the at the center (Tx), then Rx1 and Rx2 receive echoes simultaneously. The echo received by the *m*-th channel can be expressed as:

(1) $$\begin{array}{cc} s_{m}\left(\tau ,\eta \right) & =\iint \Gamma_{m}\exp(j\zeta_{m})\sigma \left(x,y\right)h\left(\tau -\frac{R_{T}(x,y,z,\eta )+R_{Rm}(x,y,z,\eta )}{c}\right)\cdot g\left(\eta -\frac{x-x_{m}}{v_{s}}\right)\\ & \cdot \exp\left\{j\pi K_{r}{\left[\tau -\left(R_{T}(x,y,z,\eta )+R_{Rm}(x,y,z,\eta )\right)/c\right]}^{2}\right\}\\ & \cdot \exp\left\{\frac{-j2\pi \left(R_{T}(x,y,z,\eta )+R_{Rm}(x,y,z,\eta )\right)}{\lambda }\right\}\mathrm{dxdy}, \end{array}$$

where $R_{T}\left(x,y,z,\eta \right)=\sqrt{{\left(x-v_{s}\eta \right)}^{2}+y^{2}+z^{2}}$ denotes the slant range of the transmitted center to the scattering point at (x,y,0). $R_{Rm}\left(x,y,z,\eta \right)=\sqrt{{\left(x-x_{m}-v_{s}\eta \right)}^{2}+y^{2}+z^{2}}$ is the slant range of the received signal of the *m*-th channel. $h\left(\tau -\frac{R_{T}(x,y,z,\eta )+R_{Rm}(x,y,z,\eta )}{c}\right)$ and $g\left(\eta -\frac{x-x_{m}}{v_{s}}\right)$ are the range antenna pattern and azimuth antenna pattern of the scattering point at (x,y,0), respectively. As the static scene is a multipoint target, $s_{m}\left(\tau ,\eta \right)$ is the accumulation of the signals of all the scattering points in the scene. 

#### 2.2.2. Signal Model of Moving Targets

The moving target is modeled as a point scatter with constant radar cross section (RCS) for simplicity. The received signal of the *m*-th channel can be expressed as:

(2) $$\begin{array}{cc} s_{m,t}\left(\tau ,\eta \right) & =\sigma_{t}\cdot h\left(\frac{\tau -\left(R_{T}(\eta )+R_{Rm}(\eta )\right)/c}{T_{p}}\right)g\left(\frac{\eta -\left(x-x_{m}\right)/v_{s}}{T_{s}}\right)\\ & \cdot \exp\left\{j\pi K_{r}{\left[\tau -\left(R_{T}\left(\eta \right)+R_{Rm}\left(\eta \right)\right)/c\right]}^{2}\right\}\cdot \exp\left\{-j\frac{2\pi }{\lambda }\cdot \left[R_{T}\left(\eta \right)+R_{Rm}\left(\eta \right)\right]\right\}, \end{array}$$

where $s_{m,t}\left(\tau ,\eta \right)$ denotes the echo of the moving target received by the *m*-th channel. $R_{T}(\eta )$ and $R_{Rm}(\eta )$ denote the slant distance of the moving target to the transmit center and the *m*-th receiver, respectively. From the geometry of the platform and the moving target in the slant-range plane in Figure 1c, $R_{T}(\eta )$ and $R_{Rm}(\eta )$ can be expressed as:

(3) $$R_{T}\left(\eta \right)=\sqrt{{\left(\left(v_{s}-v_{a}\right)\eta \right)}^{2}+{\left(v_{r}\eta +R_{0}\right)}^{2}}\approx R_{0}+v_{r}\eta +\frac{{\left(v_{s}\eta \right)}^{2}}{2R_{0}},$$

(4) $$R_{Rm}\left(\eta \right)=\sqrt{{\left(\left(v_{s}-v_{a}\right)\eta +x_{m}\right)}^{2}+{\left(v_{r}\eta +R_{0}\right)}^{2}}\approx R_{0}+v_{r}\eta +\frac{{\left(v_{s}\eta +x_{m}\right)}^{2}}{2R_{0}},$$

where $R_{0}$ is the shortest slant distance of the target. In Equations (3) and (4), $v_{a}$ can be ignored as it is far smaller than the satellite velocity $v_{s}$.

From Equations (1) and (2), the Doppler centroid of the static scene and the moving target can be expressed as (5) and (6), respectively:

(5) $$f_{dc,0}=-\frac{v_{s}x_{m}}{\lambda R_{0}},$$

(6) $$f_{dc,t}=-\left(\frac{2v_{r}}{\lambda }+\frac{v_{s}x_{m}}{\lambda R_{0}}\right).$$

It is noted in Equation (6) that the Doppler centroid of the moving target varies with the radial velocity. If conduct an imaging algorithm with the parameters of the static target in Equation (5), the Doppler centroid mismatch will cause azimuth offset of the target’s location [17].

$\phi_{c}$ and $\phi_{t}$ are the cone angles of the clutter and the moving target, respectively. From Equations (5) and (6), the existence of the target motion results in a certain offset of the Doppler frequency. For a side–looking SAR system, the relationship of the cone angle and the Doppler frequency can be expressed as [19]:

(7) $$f_{a}\left(\phi_{c}\right)=\frac{2v_{s}}{\lambda }\sin\phi_{c},$$

(8) $$f_{t,a}\left(\phi_{t}\right)=\frac{2v_{s}}{\lambda }\sin\phi_{t}+\Delta f_{t,a}\left(\phi_{t}\right)=\frac{2v_{s}}{\lambda }\sin\phi_{t}+\frac{2v_{r}}{\lambda }.$$

**Figure 3:**
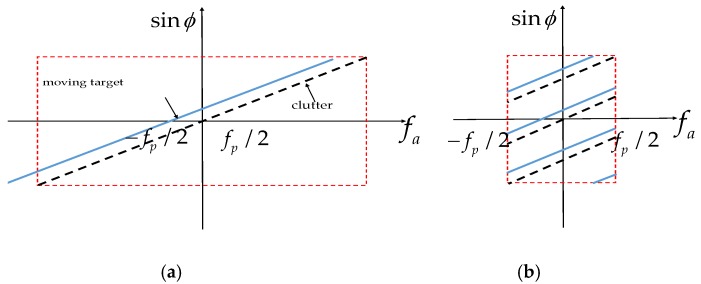
Relationship between $f_{a}$ and sinϕ: (**a**) unambiguous and (**b**) Doppler ambiguous.

Figure 3a illustrates the linear relationship between $f_{a}$ and sinϕ, where the dotted line stands for the clutter, and the solid line is the ground moving target. For a HRWS SAR system, there is under-sampling in azimuth and Doppler spectrum ambiguity for a single channel. The spatial-temporal spectra of echoes are shown in Figure 3b in practice, where the Doppler spectra of the clutter and the moving target are both folded.

To obtain unambiguous imaging for the multichannel SAR system, the echoes of all channels are sampled and recombined to get equivalent single-channel signal without Doppler spectrum ambiguities. According to the uniform sampling theory [8], the optimal PRF entailing uniform sampling meets the Displaced Phase Center Antenna (DPCA) condition:

(9) $$PRF_{uni}=\frac{2\cdot v_{s}}{M\cdot d}.$$

However, due to the inconstancy of satellite velocity and the diversity of PRF, the practical HRWS system can hardly satisfy Equation (9). The reconstruction algorithm restores the normal unambiguous data by passing the echoes through the reconstruction filter. If the motion parameters are unknown, the echoes of the moving target will be processed by the same filter as the static targets. Thus, false targets will arise as the signal of the moving target is still non-uniformly sampled in azimuth.

## 3. Processing Overview

**Figure 4:**
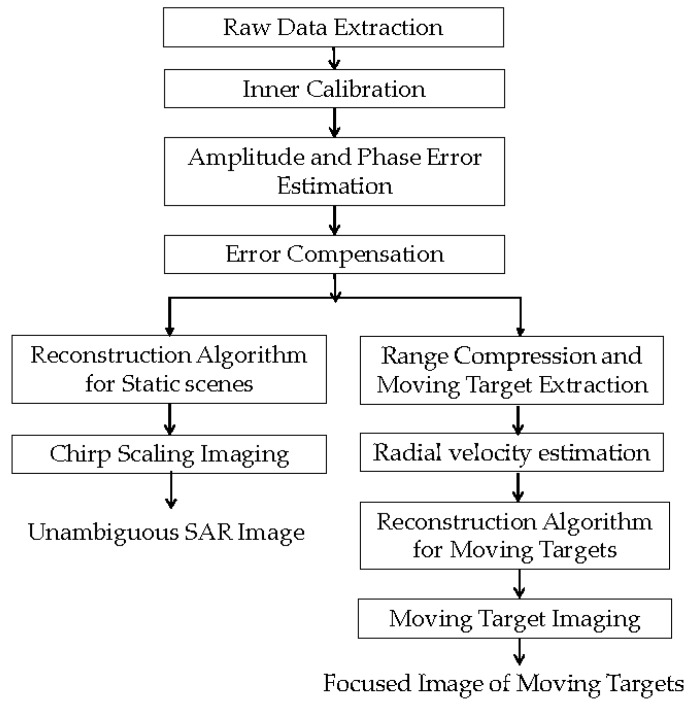
Processing flow for unambiguous imaging.

In this section, we propose a complete process of unambiguous imaging of both static scenes and moving targets. Figure 4 is the processing flow of echoes of the Gaofen-3 DRC mode. 

From Figure 4, three techniques are vital for obtaining focused images of static scenes and moving targets: amplitude and phase error estimation and compensation, reconstruction algorithm for static scenes and moving targets, and radial velocity estimation. 

### 3.1. Channel Imbalance Estimation

Mismatches in amplitude and phase possibly exist between two receive channels, the causes of which are introduced above. The amplitude and phase errors will cause degraded resolution and severe ambiguities in the SAR image. The amplitude mismatch between the two channels can be estimated by simple channel balancing:

(10) $$\Gamma_{21}≜\frac{\Gamma_{2}}{\Gamma_{1}}=\sqrt{\frac{E\left\{\left|s_{1}\left(\tau ,\eta \right)\cdot s_{2}^{*}\left(\tau ,\eta \right)\right|\right\}}{E\left\{{\left|s_{1}\left(\tau ,\eta \right)\right|}^{2}\right\}}},$$

where $\Gamma_{21}$ is the amplitude offset of channel 2 relative to channel 1. 

The phase mismatch is estimated using the OSM, which has been evaluated via simulation and applied to airborne multichannel SAR systems without spaceborne application [6]. Jin et al. [7] showed that the OSM is an effective estimator for all scenes, with no deterioration for heterogeneous areas. The OSM is based on the orthogonality between the signal subspace and noise subspaces of the sampled covariance matrix. Details of this algorithm are as follows:

*Step 1*: Conduct the Fourier transform in azimuth of the dual-channel signals in Equation (1). The sampled value is denoted as X(n):

(11) $$X(n)=G_{a}AS(n)+u(n),\;n=1,2,...,N,$$

where N is the number of Doppler bins, u(n) is the additive thermal noise. $A={\left[a_{1},a_{2}\right]}^{T}$ is the array steering vector, $G_{a}$ is a square matrix whose diagonal elements are phase errors in exponential form. Assume that channel 1 is the reference channel, then the phase error matrix $G_{a}$ can be written as:

(12) $$G_{a}=diag\left\{1,\exp\left(j\zeta_{2}-\zeta_{1}\right)\right\},$$

where diag{∙} function returns a square diagonal matrix of the elements of the vector on the main diagonal. 

*Step 2*: Compute the covariance matrix $R_{X}$ of the sampled echo, given by

(13) $$\begin{array}{cc} R_{X}(n) & =X(n)\cdot X{\left(n\right)}^{H}\\ & =\left(G_{a}\cdot A\right)R_{S}(n){\left(G_{a}\cdot A\right)}^{H}+\sigma_{n}^{2}I, \end{array}$$

where $R_{S}(n)$ is the correlation matrix of the signal in the *n*-th Doppler bin, and $\sigma_{n}^{2}$ is the average power of the thermal noise.

Then conduct eigenvalue decomposition of $R_{X}\left(n\right)$ to get the signal subspace $S_{s}$ and the noise subspace $S_{n}$. The eigen- decomposition of $R_{X}\left(n\right)$ is expressed as:

(14) $$R_{X}\left(n\right)=U\Sigma U^{H},$$

where $\Sigma =diag[\lambda_{1},\lambda_{2}]$ (the eigenvalues are sorted in descending order). Then the signal subspace $S_{s}$ is spanned by the vector in U corresponding to the larger eigenvalue $\lambda_{1}$, and the noise subspace $S_{n}$ is spanned by the vector in U corresponding to the smaller eigenvalue $\lambda_{2}$.

*Step 3*: Use the orthogonal subspace theory [6] to estimate the phase mismatch. Phase errors can be estimated by minimizing the cost function:

(15) $$J=\underset{G_{a}}{\arg\min}{\left(G_{a}\cdot A\right)}^{H}S_{n}S_{n}^{H}(G_{a}\cdot A).$$

Then the Lagrange multiplier algorithm is used to minimize the cost function. To ensure that the solution exists, the following constraint is set up:

(16) Aw=1,

where $w={\left[1,0\right]}^{T}$ meets the constraint, Combining the constraint (16) with the cost function (15), a new cost function can be obtained as follows:

(17) $$J=\underset{G_{a}}{\arg\min}\left\{{\left(G_{a}\cdot A\right)}^{H}S_{n}S_{n}^{H}(G_{a}\cdot A)+\varepsilon \left(1-Aw\right)\right\}.$$

Through derivation, the estimated phase error of channel two is:

(18) $$\zeta_{2}-\zeta_{1}={\left({\hat{G}}_{a}\right)}_{22},$$

where:

(19) $${\hat{G}}_{a}=diag\left(\frac{\Omega_{1}^{-1}w}{w^{T}\Omega_{1}^{-1}w}\right),$$

and:

(20) $$\Omega_{1}={\left(diag(A)\right)}^{H}S_{n}S_{n}^{H}\left(diag(A)\right).$$

Finally, average the estimated phase errors by N Doppler bins to improve robustness. This algorithm can estimate phase errors from the echoes of static scenes without additional calibration sources. In addition, as it only needs inversion of small scale matrixes, it is efficient. In addition, the OSM can estimate phase errors that vary with azimuth time.

### 3.2. Reconstruction Algorithm

The optimal PRF entailing uniform sampling meets Equation (9); any deviation from this PRF will result in a non-uniform sampling of the echo signals. The reconstruction filter algorithm for both the static scenes and the moving targets have been proposed and validated in References [8,9,10,11]. We give a brief expression of these algorithms.

#### 3.2.1. Static Scene Reconstruction

The reconstruction algorithm designs a reasonable pre-filter according to the signal model and restores the normal stripmap SAR data. The reconstruction filter is the inverse of the pre-filter. The reconstruction filter compensates for the phase shift caused by spatial sampling and antenna displacement, thus applies to non-uniformly sampled data.

For the dual-channel system, the pre-filter matrix for the additional shift is:

(21) $$H\left(f_{a}\right)=\left[\begin{array}{cc} H_{11}\left(f_{a}\right) & H_{12}\left(f_{a}+f_{p}\right)\\ H_{21}\left(f_{a}\right) & H_{22}\left(f_{a}+f_{p}\right) \end{array}\right].$$

For the static scenes, the pre-filter can be designed from the echoes of static signals expressed in Equation (1):

(22) $$H_{ij}\left(f_{a}\right)=\exp\left\{j\cdot \frac{\pi }{2v_{s}}\cdot d\cdot \left(f_{a}+\left(j-1\right)\cdot f_{p}\right)\cdot {\left(-1\right)}^{i+1}\right\},\;i,j=1,2.$$

The reconstruction filter P is obtained from the inverse of the pre-filter matrix, i.e.,

(23) $$P=H^{-1}.$$

After derivation, the *ij*-th element of P is expressed as:

(24) $$P_{ij}\left(f_{a}\right)=\frac{{\left(-1\right)}^{i-j}\cdot \exp\left\{j\frac{\pi }{2v_{s}}d\cdot \left(f_{a}+2^{i-1}-2(i-1)\right)\cdot f_{p}\cdot {\left(-1\right)}^{j}\right\}}{2\sin\left(\frac{\pi }{2v_{s}}\cdot d\cdot f_{p}\right)}.$$

Finally, the equivalent single-channel stripmap data is given by:

(25) $$s\left(\tau ,f_{a}\right)=s_{1}\left(\tau ,f_{a}\right)\cdot P_{1}\left(f_{a}\right)+s_{2}\left(\tau ,f_{a}\right)\cdot P_{2}\left(f_{a}\right)/\Gamma_{21}\cdot \exp\left\{-j\left(\zeta_{2}-\zeta_{1}\right)\right\}.$$

#### 3.2.2. Motion-Adapted HRWS Reconstruction

The spatial-temporal spectra of the moving targets have a linear offset from those of the static scenes, as shown in Figure 3. If the moving target signals are still handled with the conventional reconstruction filter in Equation (21), the Doppler spectra of the moving targets cannot be well recovered, which results in defocused imaging and false targets [10]. 

From the relationship of the cone angle and the Doppler frequency for static scenes and moving targets in Equations (7) and (8), the pre-filter of the moving targets can be evolved from that of static scenes in Equation (19), i.e.,

(26) $$H_{ij}\left(f_{a}\right)=\exp\left\{j\cdot \frac{\pi }{2v_{s}}\cdot d\cdot \left(f_{a}-\frac{2v_{r}}{\lambda }+\left(j-1\right)\cdot f_{p}\right)\cdot {\left(-1\right)}^{i+1}\right\},\;i,j=1,2.$$

Finally, the reconstruction filter P is obtained from the inverse of the pre-filter matrix, i.e.,

(27) $$P=H^{-1}.$$

From the reconstruction filter of the moving target, the estimation accuracy of the radial velocity has a significant influence on recovering the whole Doppler spectrum. Hence, the radial velocity should be estimated before reconstruction. 

### 3.3. Radial Velocity Estimation

After range compression of the echo of each channel, the moving targets’ signals are much stronger than those of the static ones, especially the moving ships on the sea surface. The moving target signal can be extracted for radial velocity estimation, reconstruction and focused imaging, as shown in Figure 4. In the experiment, the radial velocity is estimated using the traditional along-track interferometry (ATI) method, which is featured by high precision, low computational load, and capability of clutter suppression.

First, extract the trajectories of the moving target signals of two channels separately. Second, conduct moving target imaging with the traditional algorithm to get two images. Two SAR images of the moving target can be expressed as:

(28) $$S_{1}\left(\tau ,\eta \right)=\sigma \cdot T_{p}\cdot T_{s}\cdot \exp\left(-j\pi \cdot \frac{f_{1}^{2}}{K_{a}}\right)\cdot \sin c\left[\pi \left|K_{a}\right|T_{s}\cdot \left(\eta +\frac{f_{1}}{K_{a}}\right)\right]\cdot \sin c\left[\pi B\cdot \left(\tau -\frac{2R_{1}\left(\eta \right)}{c}\right)\right],$$

(29) $$S_{2}\left(\tau ,\eta \right)=\sigma \cdot T_{p}\cdot T_{s}\cdot \exp\left(-j\pi \cdot \frac{f_{2}^{2}}{K_{a}}\right)\cdot \sin c\left[\pi \left|K_{a}\right|T_{s}\cdot \left(\eta +\frac{f_{2}}{K_{a}}\right)\right]\cdot \sin c\left[\pi B\cdot \left(\tau -\frac{2R_{2}\left(\eta \right)}{c}\right)\right],$$

where:

(30) $$R_{1}\left(\eta \right)\approx R_{0}+v_{r}\eta +\frac{{\left(v_{s}\eta -d/2\right)}^{2}}{2R_{0}},$$

(31) $$R_{2}\left(\eta \right)\approx R_{0}+v_{r}\eta +\frac{{\left(v_{s}\eta +d/2\right)}^{2}}{2R_{0}},$$

(32) $$f_{1}=-\frac{2v_{r}}{\lambda }+\frac{d\cdot v_{s}}{\lambda R_{0}},f_{2}=-\frac{2v_{r}}{\lambda }-\frac{d\cdot v_{s}}{\lambda R_{0}}.$$

Third, estimate the radial velocity with the SAR-ATI technique.

Conjugate multiply the two complex images after image registration to obtain the interferometric phase of the target, expressed as:

(33) $$\begin{array}{cc} \phi_{ATI} & =angle\left[S_{1}\left(\tau ,\eta +\frac{f_{1}-f_{2}}{K_{a}}\right)\cdot S_{2}^{*}\left(\tau ,\eta \right)\right]\\ & =\frac{\pi }{K_{a}}\left(f_{2}^{2}-f_{1}^{2}\right)\approx \frac{4\pi d}{\lambda v_{s}}\cdot v_{r}\;. \end{array}.$$

Finally, the radial velocity of the moving target can be derived from the interferometric phase and the SAR system parameters, i.e.,

(34) $${\hat{v}}_{rATI}=\frac{\phi_{ATI}\cdot \lambda v_{s}}{4\pi d}.$$

We can obtain a well-focused image of the moving target with false target suppression and without sacrificing image resolution with the above steps.

## 4. Experimental Results 

### 4.1. Static Scene Imaging 

**Figure 5:**
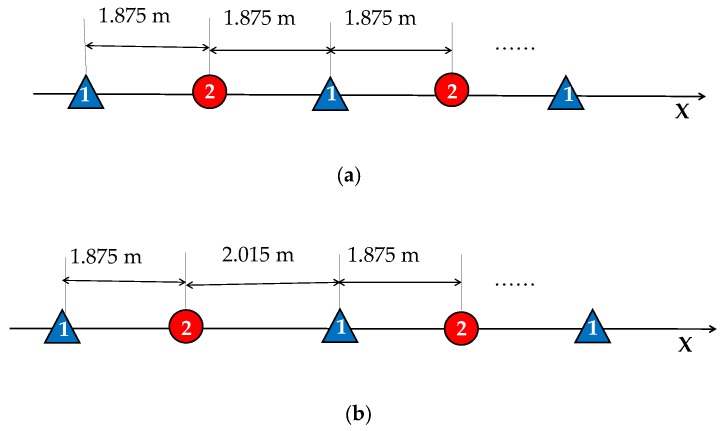
Illustration of spatial sampling: (**a**) uniform sampling and (**b**) practical sampling.

The parameters of the Gaofen-3 DRC mode are listed in Table 1. According to the satellite velocity and the aperture size, the ideal PRF satisfying uniform sampling is 2018.53 Hz. The practical 1877.1 Hz PRF leads to a separation of 2.015 m between two equivalent phase centers. The corresponding spatial sampling is illustrated in Figure 5, compared with the ideal uniform sampling. Figure 5a corresponds to the uniform sampling, where the distance between two successive spatial samples is 1.875 m. In reality, the signals are non-uniformly sampled, as shown in Figure 5b.

Here, we present the results of static scene imaging with the techniques elaborated in Section 3. We choose four typical scenes for amplitude and phase error estimation in our experiment. Scene 1 is the border region between the land and large scale sea area, scene 2 is an urban area including strong scattering points, scene 3 is the sea surface, and scene 4 is a mountainous area. The estimated amplitude and phase errors of channel 2 relative to channel 1 are summarized in Table 2. To validate the performance of the OSM, we compare the estimated phase errors with those estimated by the correlation method, which is an effective method applied in the TerraSAR-X dual-channel mode in [9].

**Figure 6:**
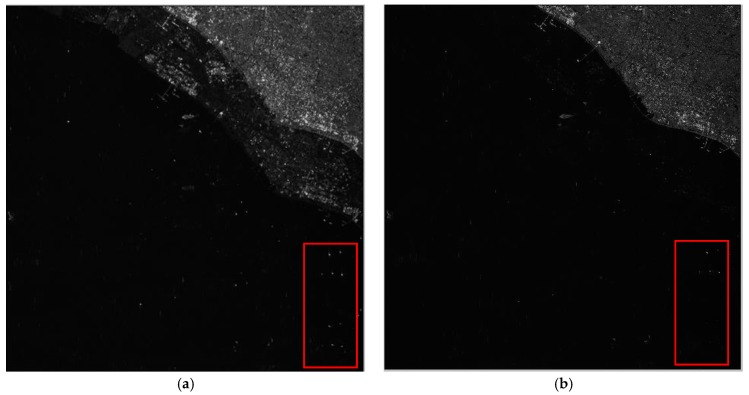
Imaging results of Scene 1: (**a**) before error compensation and reconstruction and (**b**) after error compensation and reconstruction.

**Figure 7:**
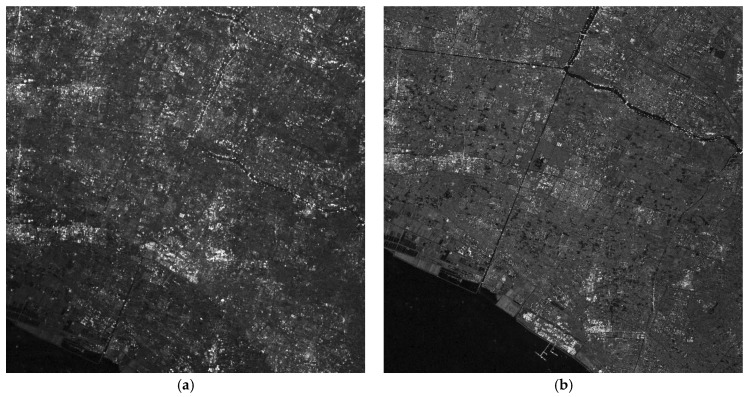
Imaging results of Scene 2: (**a**) before error compensation and reconstruction and (**b**) after error compensation and reconstruction.

**Figure 8:**
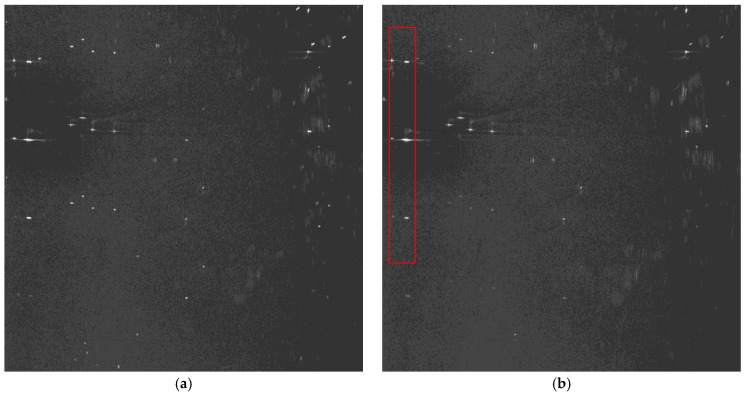
Imaging results of Scene 3: (**a**) before error compensation and reconstruction and (**b**) after error compensation and reconstruction.

**Figure 9:**
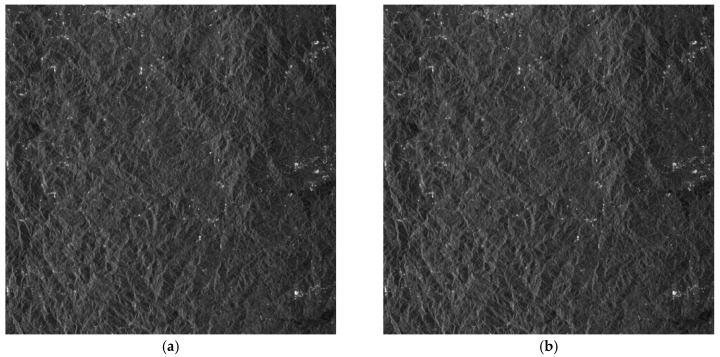
Imaging results of Scene 4: (**a**) before error compensation and reconstruction and (**b**) after error compensation and reconstruction.

After channel error estimation, we demonstrate the imaging results before and after error compensation and unambiguous reconstruction for the four areas in Figure 6, Figure 7, Figure 8 and Figure 9. As the phase errors estimated by the OSM and the correlation method are very close, there is no visual difference in the imaging results after compensation for phase errors estimated by the two algorithms. Hence, the imaging results with compensation for the phase errors by the correlation method are not given here.

From the amplitude and phase offsets in Table 2, the channel mismatches estimated by different scenes are very close, which verifies the system stability of the Gaofen-3 satellite. Moreover, the estimation performance of the OSM and the correction method are comparable, which verifies the validity of the OSM. In Figure 6a, the azimuth ambiguities of the land are rather obvious due to the weak sea clutter. On the contrary, the ambiguities are much suppressed in Figure 6b. In Figure 7a, the ambiguities of strong scattering points seriously affect the imaging quality of the urban area, and the quality is much improved with the proposed procedure in Figure 7b. Comparing Figure 8a with Figure 8b the azimuth ambiguities of the sea surface are much suppressed after error compensation and reconstruction. There are still false targets around ships targets; we can conclude that the ships are moving with some radial velocities. In Section 4.2, we will discuss velocity estimation and moving target imaging of the target ship selected in the red frame. As the scattering of the mountainous area is homogeneous, the effect of error compensation and reconstruction in Figure 9 is not visually apparent.

To quantitatively compare the imaging quality, we compute the azimuth ambiguity-to-signal ratio (AASR) of the strong scattering points. AASR means the ratio of the power of an ambiguous target to the power of a real target in the image, i.e.,

(35) $$AASR=10\cdot \log\frac{P_{A}}{P_{S}},$$

where $P_{A}$ is the power of the ambiguous target, and $P_{S}$ is the power of the real one. 

**Figure 10:**
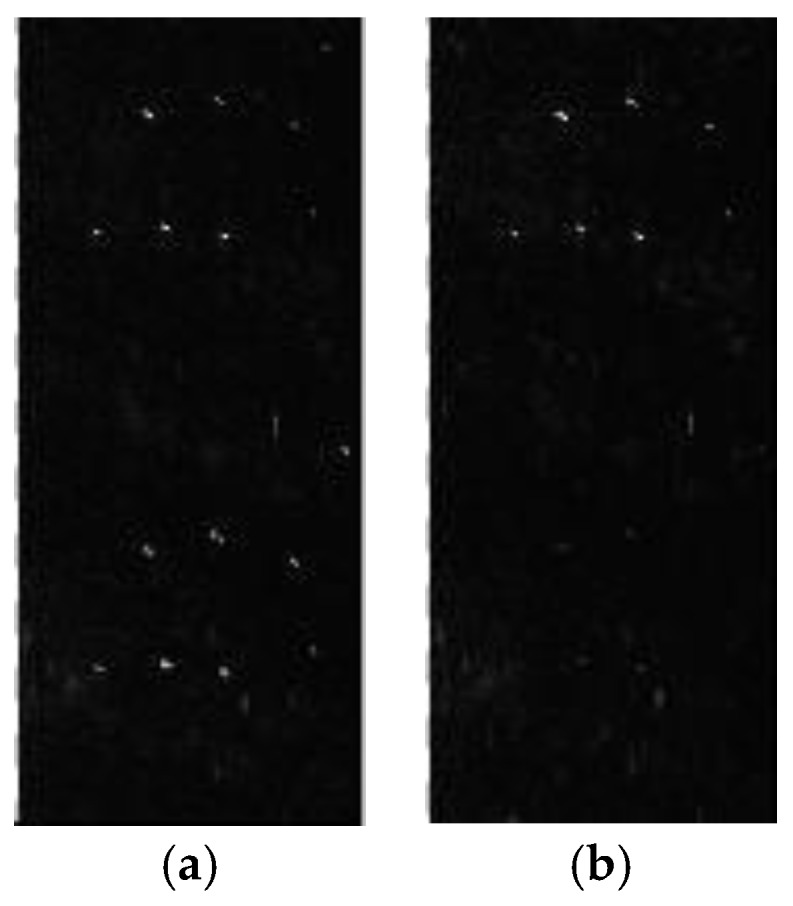
Enlargements of the selected areas in Figure 6: (**a**) corresponding to Figure 6a and (**b**) corresponding to Figure 6b.

Figure 10a,b correspond to the selected areas in Figure 6a,b, respectively. As can be seen in Figure 10a, there are five strong scattering points in the selected area. The false targets are evident in Figure 10a along the azimuth, and are suppressed in Figure 10b with the proposed algorithm. We compute the AASRs of the five targets before and after error compensation and reconstruction, respectively. Then average the AASRs of the five targets to get a mean value. 

Finally, the AASR is −15.3 dB corresponding to Figure 10a, and −35.62 dB corresponding to Figure 10b. In addition, the AASR is −35.57 dB after compensation for the phase error estimated by the correlation method. Hence, the OSM estimator combined with the reconstruction filter can achieve a remarkable improvement in the imaging quality.

### 4.2. Estimation and Imaging of Moving Targets 

**Figure 11:**
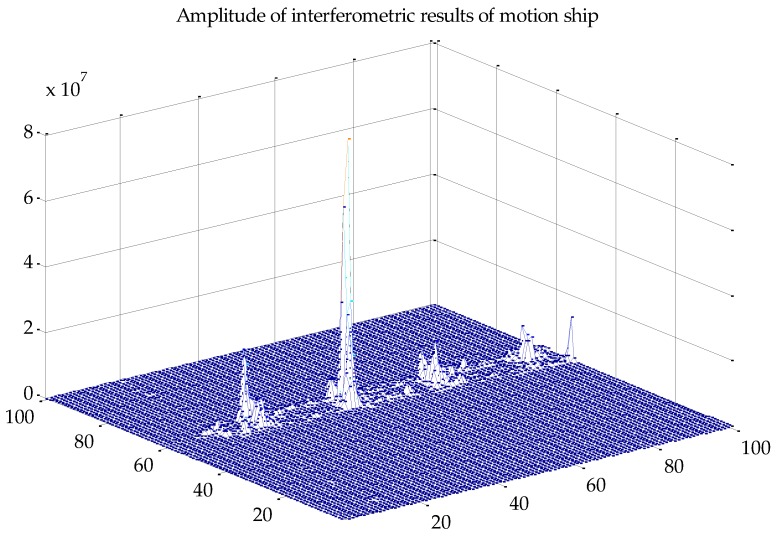
Amplitude of interferometric result of the moving ship.

We discuss velocity estimation and moving target imaging of the target ship selected in the red frame in Figure 8b. With the proposed procedure in Figure 4, the range-compressed echoes of the moving target are extracted for each channel respectively. Then the CS algorithm is conducted to obtain two images of the moving target, followed by interferometric processing. Figure 11 presents the amplitude of the interferometric result of the 100 × 100 units around the moving ship, where the clutter is suppressed with the ATI technique and the profile of the ship is obvious. With the ATI method, the estimated radial velocity is 6.37 m/s. The estimation accuracy can be weighed by comparing the imaging result of the motion-adapted reconstruction with the conventional reconstruction.

**Figure 12:**
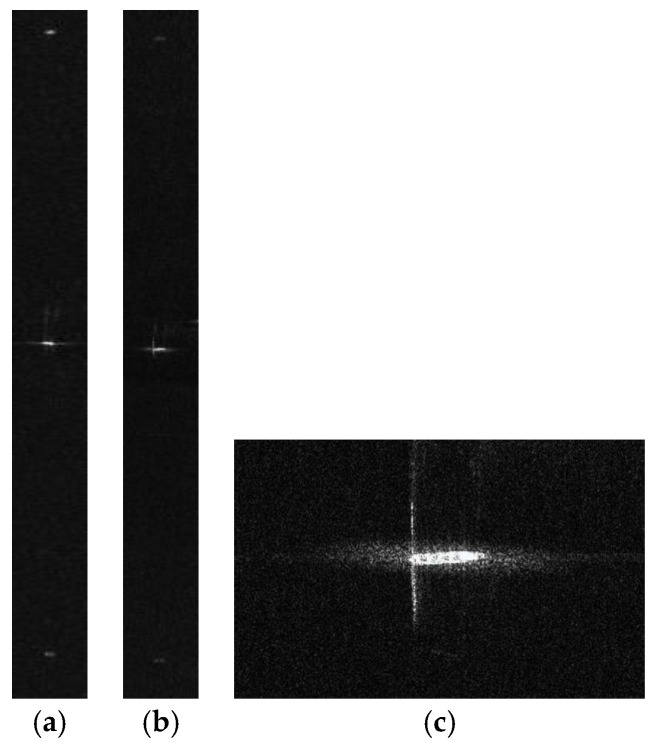
Imaging result of the moving ship: (**a**) with the conventional recontruction; (**b**) with the motion- adapted reconstruction; and (**c**) the magnified result.

**Figure 13:**
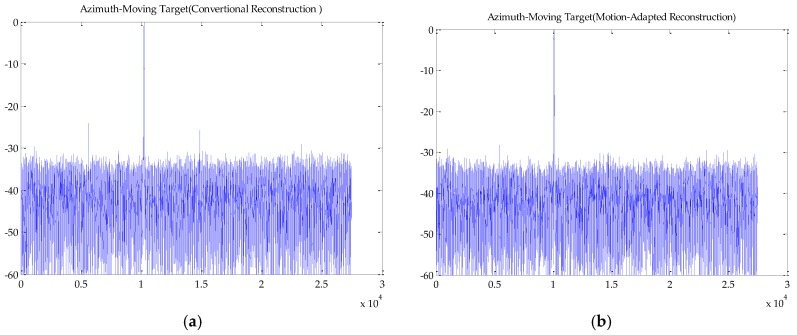
Azimuth dimensional image: (**a**) azimuth profile of Figure 12a and (**b**) azimuth profile of Figure 12b.

The motion-adapted moving target reconstruction algorithm can be applied to the extracted echoes of the moving target to obtain the unambiguous equivalent single-channel echo of the moving ship. We compare the imaging result of the motion-adapted reconstruction to that of the conventional reconstruction algorithm for static targets. The imaging results are shown in Figure 12, where Figure 12a is the moving target image with the conventional reconstruction algorithm, and the false targets are rather obvious in pairs along the azimuth. After motion-adapted reconstruction with the estimated radial velocity, the false targets are much suppressed in Figure 12b. The magnified image of the ship is presented in Figure 12c. Finally, the azimuth-dimensional imageries of the moving target are shown in Figure 13, where Figure 13a corresponds to the azimuth profile of Figure 12a, and Figure 13b corresponds to the azimuth profile of Figure 12b. The azimuth-dimensional imageries exhibit the effect of radial velocity estimation and motion-adapted reconstruction more obviously. The maximum power of false targets relative to the real one is −22.7783 dB for the convertional reconstruction algorithm. On the contrary, the false targets are submerged in clutter and noise with the proposed imaging procedure.

## 5. Discussion

The experimental results in Section 4 demonstrate an effective suppression of azimuth ambiguities for both static scenes and moving targets with the first Chinese dual-channel Spaceborne SAR sensor. From the imaging results of static scenes in Figure 6, Figure 7, Figure 8 and Figure 9, the channel error compensation and reconstruction algorithms are vital for unambiguous imaging of static scenes. The results can verify the system stability of the Gaofen-3 satellite, and validate the effectiveness of the OSM on channel error estimation for multichannel Spaceborne SAR systems.

From the results of phase error estimation and the azimuth ambiguity-to-signal ratio (AASR), the traditional correlation method and the OSM are comparable in performance. However, for the future multichannel SAR systems with more receivers, the correlation method will degrade as the correlation will reduce between a distant channel and the reference one, which is validated in [21]. The OSM is based on the orthogonality between the signal subspace and the noise subspace, so the perfomance will not degrade for multiple receive channels. 

The results in Section 4.2 explained the effect of multiple receive channels on moving target imaging. Although error compensation and reconstuction algorithm can suppress azimuth ambiguities for static scenes, false targets still exist for moving ships. With radial velocity estimation and motion-adapted reconstruction algorithm, we obtain focused imaging of the moving ship with false targets suppressed. 

With higher demands for HRWS SAR imaging, spaceborne SAR sensors with more receive channels are being developed worldwide. Experimental results demonstrate the feasibility of the OSM estimator and conventional reconstruction for static scene imaging, and the ATI estimator and motion-adapted reconstruction for moving target imaging. Hence, the proposed procedure in Figure 4 can be applied in future HRWS SAR sensors with more channels. 

## 6. Conclusions

This paper proposes an integrated unambiguous imaging scheme for both static scenes and moving targets with the first Chinese dual-channel SAR sensor. The unambiguous imaging of the static scenes mainly depends on channel error estimation and the reconstruction algorithm. The focused imaging of moving targets relies on the velocity estimation accuracy and motion-adapted reconstruction. This is the first successful application of the OSM in a spaceborne experiment of channel error estimation. Moreover, we achieved the first motion-adapted reconstruction algorithm with the spaceborne multichannel SAR system. The experimental results exhibit good suppression of azimuth ambiguities of the static scene imaging and false target elimination of moving targets. The proposed procedure has great potential for application in future HRWS SAR systems.

## Figures and Tables

**Table 1:** Imaging Parameters.

Parameter	Symbol	Value
Wavelength	λ	0.05556 m
Look Angle	θ	28.81°
PRF	$f_{p}$	1877.7 Hz
Doppler Bandwidth	$B_{a}$	2470.53 Hz
Satellite Velocity	$v_{s}$	7569.5 m/s
Sample Frequency	$f_{s}$	133.33 MHz
Bandwidth	$B_{r}$	80 MHz
Pulsewidth	$T_{r}$	54.99 us

**Table 2:** Estimated channel mismatches.

Scenes	Estimated Amplitude Error	Estimated Phase Error with the OSM (°)	Estimated Phase Error with the Correlation Method (°)
Scene 1	1.1415	14.540	14.336
Scene 2	1.1250	14.657	14.483
Scene 3	1.1777	14.494	14.320
Scene 4	1.1661	15.249	15.092
